# Efficacy and Safety of VMAT-2 Inhibitors and Dopamine Stabilizers for Huntington’s Chorea: A Systematic Review, Meta-Analysis, and Trial Sequential Analysis

**DOI:** 10.3390/medsci13030201

**Published:** 2025-09-22

**Authors:** Lautaro Manuel Floridia Rietmann, Candela Romano, Salma Alejandra Beltrán Covarrubias, Jose Antonio Gomez Miranda, Omar Enrique Briceño Cardeña, Shwetha Shenod, Ada Victoria Marrero Peralta, Genesis Mariana Ferrer Zavala, Prasanth Hanumanthu, Omar Borges Sosa, Ernesto Calderon Martinez

**Affiliations:** 1 Facultad de Ciencias Médicas, Universidad Nacional del Litoral, Santa Fe 3000, Argentina; rietmann394@gmail.com; 2 Department of Pediatrics, University of California, Irvine, CA 92697, USA; draromanocandela@gmail.com; 3 Centro Universitario de Ciencias de la Salud, Universidad de Guadalajara, Guadalajara 44340, Mexico; salmabeltranmd@gmail.com; 4 Facultad de Medicina, Universidad del del Salvador, San Salvador 1101, El Salvador; gomezjosemd85@gmail.com; 5 Facultad de Medicina, Universidad Autónoma de Yucatán, Mérida 97000, Mexico; omarrbricenomx@gmail.com; 6 PSG Institute of Medical Sciences & Research, Coimbatore 641004, India; shwetha18shenod@gmail.com; 7 Facultad de Ciencias de la Salud, Universidad Tecnológica de Santiago, Santiago de los Caballeros 51000, Dominican Republic; adamarrero02@hotmail.com; 8 Facultad de Medicina, La Universidad del Zulia, Maracaibo 4002, Venezuela; geneferrer23@gmail.com; 9 International Faculty of Medicine, Tbilisi State Medical University, Tbilisi 0186, Georgia; trueprash01@gmail.com; 10 Department of Environmental and Occupational Health, School of Public Health, Indiana University, Bloomington, IN 47405, USA; oborges@iu.edu; 11 Department of Internal Medicine, University of Texas at Houston Medical Center, Houston, TX 77030, USA

**Keywords:** chorea, efficacy, safety, Huntington’s disease, tetrabenazine, pridopidine, meta-analysis

## Abstract

Background: Huntington’s disease (HD) causes progressive motor dysfunction, with chorea as its hallmark symptom. Vesicular monoamine transporter 2 (VMAT 2) inhibitors (tetrabenazine, deutetrabenazine, valbenazine) are established symptomatic therapies, while dopamine stabilizers (pridopidine, ordopidine) are emerging therapies, but their net benefit and safety remain uncertain. Methods: Seven databases were searched through May 2025 following PRISMA guidelines. Random effects meta-analyses calculated mean differences (MDs) for the Unified Huntington Disease Rating Scale total motor score (UHDRS TMS) and total maximal chorea score (TMC), plus risk ratios (RRs) for adverse events (AEs). Trial Sequential Analysis (TSA) applied a Lan DeMets O’Brien Fleming α spending function with 80% power. Results: Seven randomized trials (1431 participants) met inclusion criteria. VMAT 2 inhibitors significantly improved motor outcomes versus placebo (UHDRS TMS: MD −3.80, 95% CI −5.76 to −1.83; TMC: MD −3.05, 95% CI −3.84 to −2.26; both I^2^ = 0%). Dopamine stabilizers produced no meaningful change (UHDRS TMS: MD −0.98, 95% CI −2.48 to 0.51; I^2^ = 32%). Neither class increased total AEs (VMAT 2: RR 1.21, 95% CI 0.99 to 1.48; dopamine stabilizers: RR 1.05, 95% CI 0.92 to 1.20; both I^2^ = 0%). TSA confirmed robust evidence for VMAT 2 benefits on TMC but indicated additional data are required to verify dopamine stabilizer effects on UHDRS TMS. Trial sequential analysis confirmed the reliability of VMAT2 for TMC; however, the sample size was insufficient to draw conclusions about the effects of dopamine stabilizers on UHDRS TMS or their safety outcomes, indicating that additional data are needed. Conclusions: VMAT-2 inhibitors may suggest potential improvements in motor symptoms in Huntington’s disease, while current evidence does not demonstrate a significant benefit of dopamine stabilizers. The safety profiles of both treatments appear generally comparable to placebo. Further rigorous and long-term studies are required to better establish their efficacy and safety.

## 1. Introduction

Huntington’s disease (HD) is an autosomal dominant neurodegenerative disorder with a worldwide prevalence of 3.92 per 100,000 people, typically presenting with a triad of symptoms affecting motor control (mainly chorea), cognition, and behavior [1,2]. The increasing number of diagnosed HD cases is largely attributable to advances in genetic testing. Additionally, greater life expectancy in the general population implies that some individuals may present with HD at older ages [3]. HD is caused by a pathological CAG repetition within the HTT gene located on chromosome 4 [4]. The HTT gene mutation leads to neuronal loss in the striatum and cortex and the formation of mutant huntingtin intranuclear inclusions, leading to a progressive, fatal disease course with a survival of 15–20 years after symptom onset [5].

A major challenge in the management of patients with HD is the lack of disease-modifying treatment. Currently, management strategies aim to alleviate a range of symptoms, including motor disturbances [1]. Chorea represents one of the most disabling manifestations of the disease, and it is characterized by brief, involuntary movements involving the trunk, face, and upper limbs, which progressively impair fundamental functions such as ambulation, speech, and swallowing [6]. FDA-approved VMAT2 inhibitors —tetrabenazine, deutetrabenazine, and valbenazine—are commonly used to manage hyperkinetic symptoms in Huntington’s disease. These agents act by depleting neuroactive peptides in nerve terminals, thereby mitigating excessive receptor stimulation. In contrast, pridopidine is an investigational novel drug currently proposed to act primarily as a Sigma-1 Receptor (S1R) agonist, rather than a D2 receptor modulator as believed. Through S1R activation, pridopidine may support neuronal survival [7,8,9,10]. In contrast, ordopidine acts as a dopamine D2 receptor antagonist [11].

Although individual studies have demonstrated the efficacy of VMAT2 inhibitors and dopamine stabilizers compared to placebo in HD chorea, a systematic review and meta-analysis assessing their efficacy and safety has not yet been performed [7,12,13,14]. Therefore, this study evaluates the effect and safety of VMAT2 inhibitors (tetrabenazine, deutetrabenazine, and valbenazine) and dopamine stabilizers (pridopidine and ordopidine) compared to oral placebo, based on improvements in chorea symptoms, disease progression, and treatment safety, considering the types and frequency of adverse events to inform clinical practice and guide long-term treatment decisions for Huntington’s disease chorea.

## 2. Methods

The present study conducted a comprehensive systematic review using the Preferred Reporting Items for Systematic Reviews and Meta-Analyses (PRISMA) reporting guidelines [15]. The protocol was registered with the International Prospective Register of Systematic Reviews (PROSPERO: CRD42025643598).

### 2.1. Types of Study

We conducted a systematic review of relevant studies published from the inception of the databases to 2025 that were available in English. We meticulously screened and analyzed randomized clinical trials (RCTs). We excluded narrative reviews, case reports, cross-sectional studies, and animal studies. Furthermore, we excluded studies that did not provide a clear description of their operationalization, were duplicated, or were unable to obtain necessary data.

### 2.2. Types of Participants

This study includes pediatric and adult participants of all genders with a genetically confirmed diagnosis of Huntington’s disease and clinically diagnosed chorea. Eligible participants may have either naive or prior exposure to VMAT-2 inhibitors or ordopidine. Exclusion criteria include individuals with other movement disorders, neuropsychiatric diagnoses, or progressive neurodegenerative conditions (e.g., dystonia, essential tremors, tic disorders, myoclonus, functional movement disorders, trauma and stressor related disorders, neurodevelopmental disorders, Alzheimer’s disease, Parkinson’s disease, amyotrophic lateral sclerosis, multiple sclerosis, spinocerebellar ataxia, spinal muscular atrophy, Lewy Body dementia, frontotemporal dementia, corticobasal degeneration, progressive supranuclear palsy, prion diseases, Leigh Syndrome, neuronal ceroid lipofuscinosis, and others). The objective is to involve a varied group of participants to broaden insights regarding the impact of the intervention.

### 2.3. Types of Intervention

This study will evaluate the effect of VMAT-2 inhibitor drugs (valbenazine, tetrabenazine, deutetrabenazine) and dopamine stabilizers (pridopidine or ordopidine) in our population by comparing them with oral placebo. To maintain the integrity of the study, the following medications will not be included: VMAT-2 inhibitors or dopamine stabilizers used for other neurodegenerative conditions than Huntington’s chorea, VMAT-2 inhibitors or dopamine stabilizers used combined with other drugs such as antipsychotics, anticonvulsants, and benzodiazepines.

### 2.4. Outcomes

The standard clinical assessment tool for Huntington’s disease is the Unified Huntington’s Disease Rating Scale (UHDRS), which demonstrates high reliability in motor scores and was consistently used in the included studies to assess treatment efficacy. This tool was selected because it is the most widely validated, sensitive, and recommended measure of motor impairment in Huntington’s disease compared with other scales, enabling consistent comparisons across studies.

### 2.5. Searching Methods

A systematic search was initially conducted on 26 January 2025 on PubMed, Web of Science, Embase, CINAHL, Scopus, Cochrane, and CINK using the following terms: ‘’Huntington’s disease’’, ‘’Huntington’s chorea”, dopaminergic stabilizers, VMAT-2 inhibitors, valbenazine, tetrabenazine, deutetrabenazine, pridopidine, ordopidine. All detailed search strategies can be found in the Supplementary Material, Tables S1–S7.

### 2.6. Selection of Studies

All references were exported to Rayyan [16] and duplicates were removed. Two authors independently completed the eligibility assessment, first by title and abstract analysis and, afterward, by full-text assessment. Disagreements between reviewers were resolved through discussion with a third reviewer.

### 2.7. Data Extraction

Data extraction was conducted by four independent reviewers, with discrepancies resolved by consensus. If multiple overlapping reports were found, the most relevant or earliest published report was prioritized. The extracted variables included study details (author, year, title, DOI, country, design), genetic data (CAG repeats in HTT gene), demographics (sample size, age, follow-up duration), treatment (drug type, dosing, duration), and clinical outcomes (UHDRS Total Motor Score (TMS) and Total Motor Chorea (TMC), changes, treatment differences). Adverse events, including serious events, drug discontinuation, and specific side effects (e.g., diarrhea, falls, headache, depression, Parkinsonism) were also recorded. We had planned to contact study authors if relevant data were missing. However, all prespecified outcomes were fully reported in the included studies and no additional author contact was required.

### 2.8. Assessment of Risk of Bias

Two independent reviewers assessed the methodological quality of the included studies using the Cochrane Risk of Bias 2.0 (ROB2) tool [17]. Any disagreements were resolved through discussion with a third author.

### 2.9. Statistical Analysis

A meta-analysis was performed using R version 3.4.3 [18]. The pooled effect of the outcomes was examined using a random-effects meta-analysis (DerSimonian–Laird approach) [19] number of studies reporting an outcome of interest was insufficient, only a qualitative analysis of the results was performed. Effect sizes were expressed as Relative risk (RR) or mean difference (MD) and 95% confidence interval. The I^2^ statistics assessed heterogeneity and the following cut-off values used for interpretation: <25, 25–50, and >50% were considered small, medium, and large heterogeneity, respectively. Whenever possible, sensitivity analyses according to the leave-one-out method were performed to determine the influence of individual studies on the overall effect. Egger’s regression test was used to examine publication bias when 10 or more reports with the same outcome were available. Subgroup analyses were performed for primary outcomes. Additionally, Trial Sequential Analysis (TSA) was conducted using the Copenhagen Trial Unit TSA software (version 0.9.5.10 Beta) to assess the conclusiveness of statistically significant results and to determine whether inconclusive findings necessitated additional sample size [20]. TSA was applied to both dichotomous and continuous outcomes, with RR used for dichotomous outcomes and MD for continuous outcomes. The Lan-DeMets flexible O’Brien-Fleming alpha-spending function was employed to control for Type I error, using a two-sided significance threshold of 5%. To minimize Type II error, the power was set at 80% (Type II error = 20%). The Required Information Size (RIS) was estimated based on expected effect sizes and event rates, and when appropriate, an event size-based information axis was used. TSA monitoring boundaries were utilized to determine if sufficient evidence had been reached, if futility could be declared, or if further studies were required.

## 3. Results

### 3.1. Study Selection

**Figure 1:**
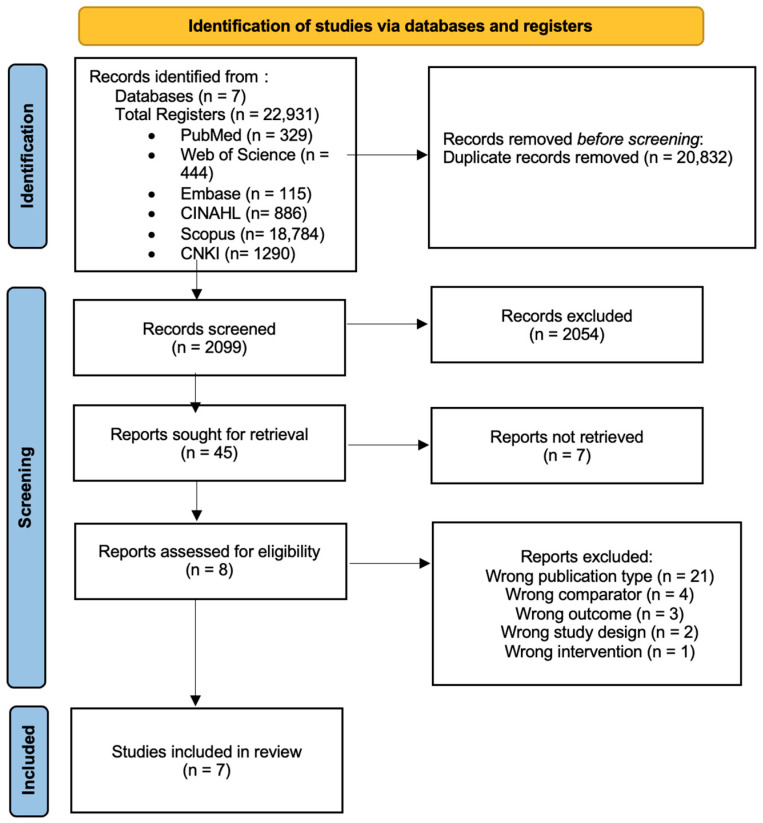
PRISMA flow chart. This flow diagram outlines the systematic process of identifying, screening, and including studies in this meta-analysis.

In our initial search, we identified a total of 22,931 potential articles from seven databases. After eliminating 20,832 duplicate records, we proceeded with a title and abstract screening, which led to the exclusion of 2054 articles. Of the 45 articles selected for full-text retrieval, 7 were unavailable, leaving 38 articles for further eligibility assessment. Following eligibility screening, we excluded 21 due to wrong publication type (reviews, conference abstracts, protocols, trial registrations, etc.), 4 for the wrong comparator (studies that used active drug rather than placebo as control), 3 for wrong outcome (studies without any mention of UHDRS or other validated HD motor endpoints, no reports of safety outcomes, or/and incomplete baseline or endpoint outcome reporting), 2 for incompatible study design (studies with insufficient methodological/operational descriptions or data presentation), and 1 for wrong intervention (studies mentioning use drugs outside of prespecified drug classes or studies mentioning pharmacokinetic/pharmacodynamic effects only). As a result, seven [14,21,22,23,24,25,26] studies were ultimately included in this review. A detailed summary of this process is provided in the PRISMA flow chart (Figure 1).

### 3.2. Study Characteristics and Participants

The seven [14,21,22,23,24,25,26] studies included a total of 1431 participants. Most of the studies were conducted in the United States and Canada (43%; *n* = 3), with the United States alone accounting for one study (14%; *n* = 1). Additionally, three studies were multinational investigations: (43%; *n* = 3). The primary outcomes assessed included the improvement of chorea symptoms measured by UHDRS scales, along with the incidence of side effects and severe adverse events. Five of the seven studies used the UHDRS TMS scale while three used the UHDRS TMC scale to measure the improvement in chorea. Of the three studies included (42.8%) evaluating pridopidine, an S1R agonist, found no significant effect on motor symptoms based on UHDRS TMS scores. Two studies (28.5%) found that VMAT-2 inhibitors had a modest effect on reducing motor symptoms, as measured by UHDRS TMS scores; however, the results were not statistically significant. Additionally, three studies (42.8%) showed that VMAT-2 inhibitors reduced motor symptoms in Huntington’s disease, as measured by the UHDRS TMC score; however, the results were not statistically significant. Studies with pridopidine (58%) reported falls as the most common adverse event, while VMAT-2 studies (42%) reported somnolence as the most frequent adverse event. There were multiple serious adverse events that varied between the groups. A more comprehensive report of this information can be found in the general outcomes (Table 1).

### 3.3. Risk of Bias Within Studies

**Figure 2:**
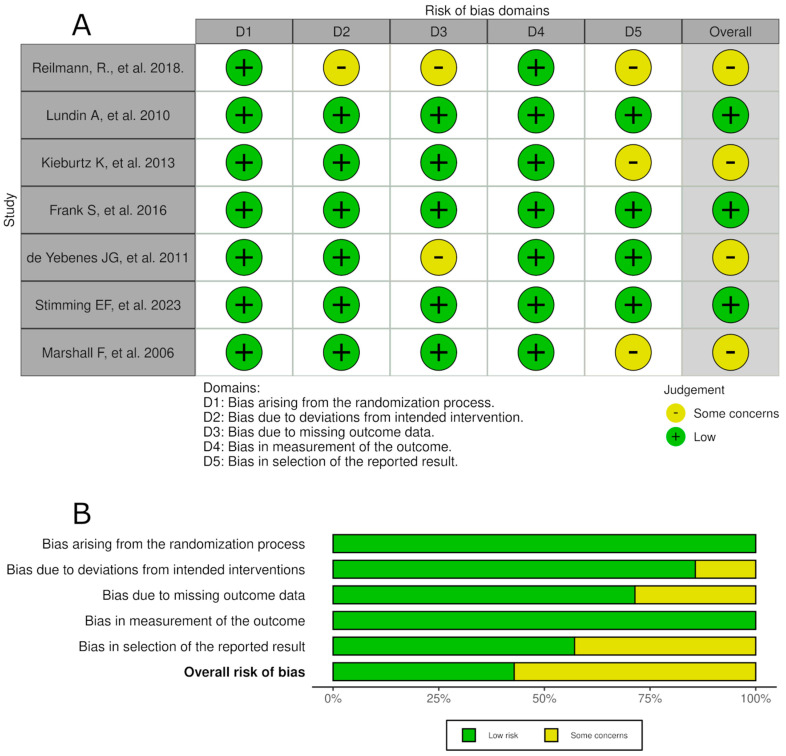
Risk of bias. Risk of bias assessment of the included randomized controlled trials using the Cochrane RoB 2.0 tool. (**A**) Risk of bias by domain for each study and (**B**) overall risk of bias presented as a percentage. Among the seven studies assessed [14,21,22,23,24,25,26], almost 50% raised some concerns, while 43% had a low risk of bias. No studies were classified as high risk.

We assessed the quality and risk of bias in the seven included randomized controlled trials using the Cochrane Risk of Bias 2.0 (ROB2) tool. Our findings show that four studies (57%) raised some concerns regarding bias, three of them in the selection of the reported results, while the remaining three studies (43%) exhibited a low risk of bias (Figure 2). Overall, our analysis indicates that all studies had either a low risk of bias or raised some concerns, with none classified as having a high risk of bias.

### 3.4. Meta Analysis

#### 3.4.1. Change in the UHDRS TMS from the Baseline: Dopamine Stabilizers

**Figure 3:**
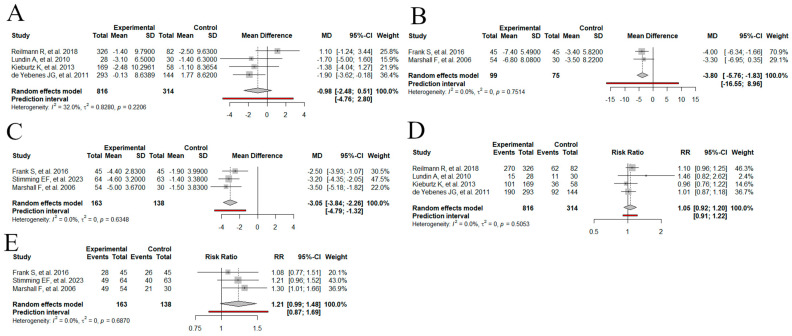
Meta-analysis forest plots. (**A**) Meta-analysis evaluating the efficacy of dopamine stabilizers (pridopidine) vs. placebo in UHDRS TMS, (**B**) the efficacy of VMAT-2 inhibitors vs. placebo in improving UHDRS TMS, (**C**) the efficacy of VMAT-2 inhibitors compared to placebo in improving UHDRS TMC, (**D**) the safety of dopamine stabilizers (pridopidine) compared to placebo analyzing reported adverse events, and (**E**) the safety of VMAT-2 inhibitors compared to placebo analyzing total reported adverse events. Analyses are based on data from the randomized controlled trials included in the meta-analysis [14,21,22,23,24,25,26].

**Figure 4:**
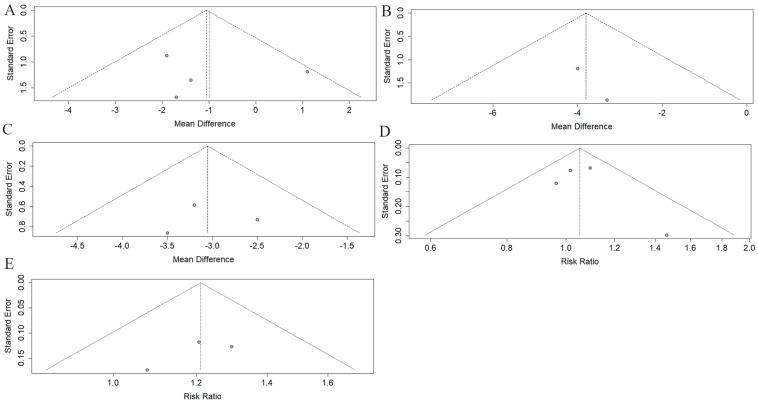
Funnel plots for publication bias. Funnel plot analysis for assessing publication bias in (**A**) the efficacy of dopamine stabilizers (pridopidine) vs. placebo in UHDRS TMS, (**B**) the efficacy of VMAT-2 inhibitors vs. placebo in improving UHDRS TMS, (**C**) the efficacy of VMAT-2 inhibitors compared to placebo in improving UHDRS TMC, (**D**) the safety of dopamine stabilizers (pridopidine) compared to placebo analyzing reported adverse events, and (**E**) the safety of VMAT-2 inhibitors compared to placebo analyzing total reported adverse events.

**Figure 5:**
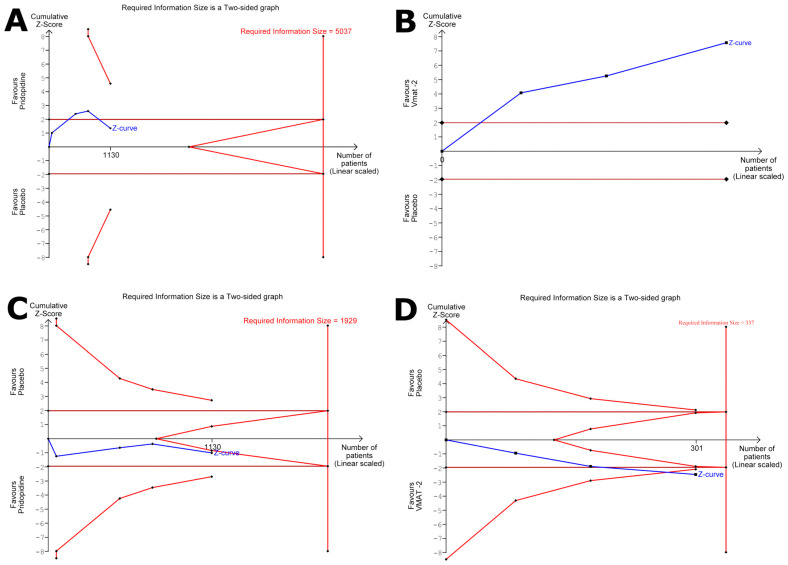
Trial sequential analysis. of (**A**) UHDRS TMS dopamine stabilizers, (**B**) UHDRS TMC VMAT-2 inhibitors, (**C**) adverse events in dopamine stabilizers, and (**D**) adverse events in VMAT-2 inhibitors.

We conducted a meta-analysis to evaluate the efficacy of dopamine stabilizers versus placebo in the UHDRS TMS scale (Figure 3A). We incorporated four studies with a total sample size of 1130 subjects, comprising 816 individuals treated with dopamine stabilizers (pridopidine, S1R agonist) and 314 in the control group treated with placebo. Using the random effects model, the pooled MD was estimated at −0.98 (95% CI: −2.48 to 0.51, I^2^ = 32%, *p* = 0.22). The funnel plot revealed asymmetry suggestive of potential publication bias (Figure 4A). Subgroup analysis was conducted to explore potential sources of heterogeneity based on country, year, risk of bias, study design, dosing escalation, and treatment period (Supplementary Tables S8–S13). For the treatment period, the random-effects model revealed a significant difference between subgroups (*p* = 0.03). Treatments lasting ≤ 26 weeks showed a significant reduction in UHDRS TMS compared to placebo (MD = −1.74, 95% CI: −3.07 to −0.42), whereas treatments lasting ˃ 26 weeks had a more uncertain effect (MD = 1.10, 95% CI: −1.24 to 3.44; Supplementary Table S13). Sensitivity analysis using the leave-one-out method identified two studies (Reilmann R et al., 2019 and Yebenes JG et al., 2011) as contributors to the observed heterogeneity (Supplementary Table S14) [22,23]. This suggests that these studies may have significantly influenced the overall meta-analysis results. Due to the limited number of remaining studies, an analysis excluding these articles was not performed. The TSA was conducted to determine whether the RIS for the UHDRS TMS was met and to assess whether further research is necessary. The RIS was 5037 patients, while the current sample size reached 1130 patients, indicating that the required sample size has not yet been achieved. The cumulative Z-curve shows a positive direction favoring the intervention but has not crossed the statistical significance boundary. Since the required sample size has not been reached and the Z-curve remains within the monitoring boundaries, the current evidence is not sufficient to confirm a definitive effect or to conclude futility (Figure 5A).

#### 3.4.2. Change in the UHDRS TMS from the Baseline: VMAT-2

We conducted a meta-analysis to evaluate the efficacy of VMAT-2 versus placebo in the UHDRS TMS scale (Figure 3B). We incorporated two studies with a total sample size of 174 subjects, comprising 99 individuals treated with VMAT-2 and 75 in the control group treated with placebo. Using a random effects model, the pooled MD was estimated at −3.80 (95% CI: −5.76 to −1.83, I^2^ = 0%, *p* = 0.75). The prediction interval ranged from −16.55 to 8.96. The funnel plot revealed asymmetry suggestive of potential publication bias (Figure 4B). The TSA was not conducted due to the low number of studies.

#### 3.4.3. Change in the UHDRS TMC from the Baseline: VMAT-2

We conducted a meta-analysis to evaluate the efficacy of VMAT-2 versus placebo in the UHDRS TMC scale (Figure 3C). We incorporated three studies with a total sample size of 301 subjects, comprising 163 individuals treated with VMAT-2 and 138 in the control group treated with placebo. Using a random effects model, the pooled MD was estimated at −3.05 (95% CI: −3.84 to −2.26, I^2^ = 0%, *p* = 0.63). The prediction interval ranged from −4.79 to −1.32. The funnel showed symmetry, ruling out possible publication bias (Figure 4C). The TSA for the UHDRS TMC was performed to evaluate whether the RIS was achieved and if the observed effect is conclusive. The cumulative sample size exceeded the RIS from the outset. This indicates that the available evidence already surpasses the predefined assumptions. The cumulative Z-curve exhibits an upward trend favoring the intervention, indicating a potential beneficial effect (Figure 5B).

#### 3.4.4. Total Adverse Events: Dopamine Stabilizers

We conducted a meta-analysis to evaluate the safety of dopamine stabilizers versus placebo with the total reported adverse events (Figure 3D). We incorporated four studies with a total sample size of 1130 subjects, comprising 816 individuals treated with dopamine stabilizers (pridopidine, S1R agonist) and 314 in the control group treated with placebo. Using a random effects model, the pooled RR was estimated at 1.05 (95% CI: 0.92 to 1.20, I^2^ = 0%, *p* = 0.50). The funnel plot revealed asymmetry suggestive of potential publication bias (Figure 4D). The TSA was performed to determine whether the RIS had been reached and to assess the conclusiveness of the results. The RIS was 1929 patients, with sequential monitoring boundaries set for benefit, harm, and futility. The cumulative Z-curve did not cross any monitoring boundaries and the required information size was not reached. With only 1130 patients included, the current evidence remains inconclusive, highlighting the need for further adequately powered trials (Figure 5C).

#### 3.4.5. Total Adverse Events: VMAT-2

We conducted a meta-analysis to evaluate the safety of VMAT-2 versus placebo in the total reported adverse events (Figure 3E). We incorporated three studies with a total sample size of 301 subjects, comprising 163 individuals treated with VMAT-2 and 138 in the control group treated with placebo. Using a random effects model, the pooled RR was estimated at 1.21 (95% CI: 0.99 to 1.48, I^2^ = 0%, *p* = 0.68). The prediction interval ranging from 0.87 to 1.69. The funnel plot revealed asymmetry suggestive of potential publication bias (Figure 4E). TSA was performed to assess whether the RIS had been reached and to evaluate the need for further research on adverse effects of VMAT2 inhibitors. The estimated RIS was 337 patients, with sequential monitoring boundaries defined for benefit, harm, and futility. The cumulative Z-curve crossed the boundary for benefit, suggesting strong evidence in favor of the intervention. However, because the RIS was not achieved, the available evidence remains underpowered (Figure 5D).

The Egger test was not carried out in all variables due to the low number of studies included. Subgroup analysis and sensitivity analysis were not performed in the last three variables due to low heterogeneity.

## 4. Discussion

Our systematic review and meta-analysis evaluated the efficacy and safety of VMAT-2 inhibitors (tetrabenazine, deutetrabenazine, valbenazine) and dopamine stabilizers (pridopidine) in treating chorea in Huntington’s disease. Dopamine stabilizers showed a slight non-statistically significant effect on UHDRS TMS (MD: −0.98, 95% CI: −2.48 to 0.51, *p* = 0.22), and TSA indicated a gradual beneficial trend, suggesting that more studies are needed to reach statistical significance. Subgroup analysis suggested that shorter treatment durations may be associated with greater motor benefit. However, this finding is exploratory and should be interpreted cautiously, as the longer-duration subgroup was based on a single study. VMAT-2 inhibitors were associated with modest reductions in motor symptoms. The meta-analysis showed a benefit on UHDRS-TMC (MD: −3.05, 95% CI: −3.84 to −2.26), confirmed by TSA, and a slightly larger but less certain effect on UHDRS TMS (MD: −3.80, 95% CI: −5.76 to −1.83), for which TSA could not be performed due to the limited number of studies.

Regarding safety, pridopidine appeared well-tolerated, with no significant increase in adverse events compared with placebo (RR = 1.05, 95% CI: 0.92 to 1.20, *p* = 0.50). However, TSA indicated that the required information size (RIS = 1929) was not met, meaning further studies are needed to confirm its safety profile. For VMAT-2 inhibitors, there was a trend toward a higher risk of adverse events (RR = 1.21, 95% CI: 0.99 to 1.48), but this did not reach statistical significance. TSA for VMAT-2 adverse events (RIS = 337) indicated that the current evidence is inconclusive and additional studies are required.

Given these findings, the selection of studies was crucial for the interpretation of results. Our meta-analysis included three Huntington Study Group trials with minimal risk of patient overlap, as each study employed distinct exclusion criteria and timelines: Marshall et al. (2006) [21], Kieburtz et al. (2012) [25], and Frank et al. (2022) [12] with stricter exclusion criteria. Although complete exclusion of patient overlap was not possible due to the lack of individual-level data despite contacting the original authors, these measures minimized the potential for duplication. Similarly, previous meta-analyses, including that of Chen S. et al. (2021) [7], did not show significant improvement in TMS with pridopidine. Key differences between their study and ours lie in the data used; they relied on primary efficacy analysis, while we used Intent-to-Treat (ITT) population data for both efficacy and safety. Chen S. et al. observed improvements in UHDRS-mMS, which excludes chorea, while our study focused on chorea as the primary efficacy measure [27]. Furthermore, our study included both VMAT-2 inhibitors and dopamine stabilizers, offering a broader evaluation of treatment effects. While VMAT-2 inhibitors showed a significant improvement in UHDRS TMC, their effect on UHDRS TMS remained inconclusive, with no heterogeneity but potential publication bias. Regarding safety, our analysis found no significant difference in adverse effects between pridopidine and placebo (RR = 1.03, 95% CI: 0.94–1.13, *p* = 0.49), aligning with previous meta-analysis [28]. On the other hand, TSA of VMAT-2 inhibitors indicated that further research is required to establish their efficacy and safety. In contrast with previous meta-analyses, the TSA of dopamine stabilizers suggested that the accumulated evidence is sufficient, and additional findings are needed.

Building on these findings or results on VMAT-2 inhibitors in Huntington’s chorea aligns with partially existing literature on their use in other neuropsychiatric conditions, such as schizophrenia. For example, a meta-analysis on tetrabenazine for psychotic disorders [29] showed modest symptom reduction. While the clinical endpoints differ—motor symptoms in HD vs. positive symptoms in schizophrenia—the directionality of benefit was similar. However, these effects did not consistently extend to broader domains such as cognition or global functioning in either context. In both cases, VMAT-2 inhibitors reduced motor symptoms in Huntington’s chorea and positive symptoms in schizophrenia, but their effects on broader outcomes were limited and often statistically insignificant. For instance, the psychosis meta-analysis noted that tetrabenazine improved positive symptoms but had little effect on cognitive and negative symptoms, reflecting our finding that motor improvements in Huntington’s chorea did not translate to overall disease progression or non-motor symptoms.

Regarding safety, while sedation and somnolence were commonly reported across conditions, it is important to note that not all VMAT-2 inhibitors are pharmacologically equivalent. Deutetrabenazine and valbenazine have been associated with improved tolerability and reduced neuropsychiatric side effects compared to tetrabenazine, likely due to differences in half-life and CNS penetration. Therefore, while mechanistic overlap may exist, clinical application and safety interpretation must be tailored to specific agents and patient populations. Differences in underlying pathophysiology—striatal degeneration in Huntington’s versus cortical-subcortical dysregulation in schizophrenia—further limit direct comparisons of efficacy or safety profiles across indications.

Although our meta-analysis combined both VMAT-2 inhibitors and dopamine stabilizer agonists, it is important to acknowledge that their inclusion in the same framework stems from clinical considerations, not pharmacological equivalence. VMAT-2 inhibitor trials were primarily designed and powered to evaluate chorea outcomes, whereas trials involving pridopidine often used broader functional endpoints. This heterogeneity in trial design may account for differences in effect size and should be considered when interpreting pooled analyses.

Our analysis underscores the distinction between VMAT-2 inhibitors and pridopidine, both in terms of pharmacologic action and trial design. VMAT-2 inhibitors directly reduce dopamine release by inhibiting vesicular transport, thereby improving hyperkinetic movements like chorea [30]. In contrast, pridopidine is now believed to exert its effects primarily via Sigma-1 receptor agonism, influencing cellular stress pathways and potentially offering neuroprotective benefits [31]. Mechanistic differences may explain the lack of motor symptom improvement observed with pridopidine in trials not powered for chorea outcomes.

Additionally, while we pooled VMAT-2 inhibitors for analytical purposes, we acknowledge important differences among tetrabenazine, deutetrabenazine, and valbenazine. Newer agents (e.g., deutetrabenazine, valbenazine) may have more favorable side effect profiles than tetrabenazine due to improved pharmacokinetics and CNS tolerability.

Furthermore, the use of pridopidine in this analysis must be interpreted with caution, given that its proposed mechanism of action has evolved. While initially categorized as S1R agonists, current evidence suggests pridopidine primarily acts through Sigma-1 receptor activation. This mechanism may not directly impact chorea symptoms, explaining the absence of significant motor improvement in several trials.

In light of these comparisons and the complexity of the findings, it is important to emphasize that our systematic review and meta-analyses are the first to comprehensively evaluate the efficacy and safety of both VMAT-2 inhibitors (tetrabenazine, deutetrabenazine, and valbenazine) and dopamine stabilizers (pridopidine and ordopidine) specifically in Huntington’s chorea. Unfortunately, no eligible manuscripts evaluating ordopidine met the inclusion criteria; therefore, the findings of this study primarily reflect the effects of S1R activation by pridopidine. While previous studies have examined these treatments individually, there has been limited research directly comparing these novel drugs against placebo in this patient population. By addressing this gap, our study provides valuable insights that could inform future treatment strategies and clinical practice in managing Huntington’s chorea, particularly in tailoring therapies to the motor and non-motor symptoms of affected individuals.

### Limitations and Practical Implications

Including English publications may lead to a non-consideration of relevant RCTs in other languages. The studies performed only in the US, Canada, and Europe limit the generalizability. Our findings suggested that VMAT-2 inhibitors, approved for HD-associated chorea, offer modest improvements in motor symptoms and should be tailored to individual patient needs, monitoring for side effects. S1R agonists did not show significant improvements in motor symptoms, but they may show promise for managing non-motor symptoms, including cognitive and psychiatric. While the results are promising, these therapies must not be a solution. Consider part of a treatment plan, including patient factors such as age, disease stage, comorbidities, and response to other medications. Our study suggests that further research is essential, with extensive trials and extended follow-up periods, to define these potential drugs and their safety profiles. Existing knowledge indicates that clinicians should guide treatment decisions by carefully considering the risks and benefits.

Adverse event reporting varied across studies and many did not clearly differentiate between mild, moderate, and severe events. This limits the ability to assess the comparative tolerability of these medications. Notably, Parkinsonism-like symptoms are a known side effect of VMAT-2 inhibitors and should be closely monitored in clinical practice. We were also unable to stratify adverse events by severity due to limited or inconsistent reporting in the source trials. Consequently, mild side effects such as headache and diarrhea were pooled alongside more severe events such as aspiration pneumonia or seizures. This limitation affects the granularity of the safety interpretation. Although adverse event rates were compared with placebo, the inability to stratify by severity limits conclusions regarding tolerability. Future trials should report AE by grade to clarify differences in clinical relevance.

The UHDRS-TMC is a subscore of the TMS scale that specifically captures chorea-related motor changes. We analyzed TMC independently to evaluate whether chorea severity responded to treatment even when broader motor impairment (TMS) did not show significant change. This distinction was especially relevant given that VMAT-2 studies were powered to detect chorea improvement, while pridopidine trials focused on functional or global endpoints. This separation is clinically relevant because TMC, as a direct measure of chorea, may detect improvements that broader scales, such as TMS, might miss due to their inclusion of rigidity, bradykinesia, and oculomotor items that may be unaffected by the interventions under study. We selected the UHDRS because it is the most widely validated and standardized clinical tool for assessing motor and functional outcomes in Huntington’s disease trials, and it has been consistently used in previous RCTs.

Finally, due to the limited number of studies available for some outcomes, especially regarding pridopidine, further data will be essential to confirm or challenge current interpretations. Unfortunately, no eligible manuscripts evaluating or dopamine met the inclusion criteria. Although author correspondence suggests a low likelihood of participant overlapping in the pridopidine trial, dual enrollment cannot be excluded because IDs were de-identified. Large-scale, multi-center RCTs with harmonized endpoints and extended follow-up are needed to inform clinical practice more robustly. Due to inconsistent reporting and heterogeneous dosing across trials, a dose-response analysis was not feasible and should be explored in future studies.

## 5. Conclusions

This meta-analysis indicates that VMAT-2 inhibitors may be associated with modest improvements in chorea symptoms among patients with Huntington’s disease, although the evidence remains limited. In contrast, pridopidine has not demonstrated a significant benefit for motor symptoms to date. Both treatments had a comparable safety profile to placebo, with no significant increase in total adverse events. However, close monitoring is recommended for patients receiving VMAT-2 inhibitors to ensure early detection and management of potential adverse effects. Despite these findings, the small sample size and limited number of studies included restricted our analysis. Future research should focus on evaluating the long-term efficacy and safety of these medications in larger, more diverse populations, as well as assessing the impact of early intervention strategies on quality of life.

## Figures and Tables

**Table 1:** General outcomes.

Author, Year	Risk of Bias	Median Age, Years	Sample Size	Follow-Up Period (Weeks)	Intervention Group and Dosage	Primary Outcomes Measured	Most Common Adverse Event	Most Common Serious Adverse Event	Treatment Discontinuation Because of ADR
Reilmann R, et al., 2018 [22]	Low	Cases: 51.9 (11.8) Control: 50.3 (11.3)	164	54	Dopamine stabilizer (pridopidine) 45 mg, 67.5 mg, 90 mg, and 112.5	UHDRS- TMS; Q-test; CGI	Falls Cases: 19 (23%) Controls: 17 (21%)	Cases: Suicidal ideation: 1 (1.2%) and aspiration pneumonia: 1 (1.2%)	Yes
Lundin A, et al., 2010 [24]	Low	Cases: 50.2 (7) Control: 56 (10)	58	NA	Dopamine stabilizer (pridopidine) 50 mg	UHDRS TMS and mMS; Raitan Trail-Making Test; Stroop Test;HADS, CGIC	Cases: tiredness: 5 (17.8%), and falls and injuries: 5 (21%) Control: infections 5 (21%).	Cases: pneumonia 1 (3.3%)	No
Kieburtz K, et al., 2013 [25]	Low	Cases: 54.3 (11) Control: 50.4 (10.5)	114	14	Dopamine stabilizer (pridopidine) 10 mg, 22.5 mg, and 45 mg initial dose 20 mg, 45 mg, and 90 mg target dose	UHDRS TMS; TFC	Falls 10 mg: Cases: 9 (16.1%) Control: 7 (12.1%) 22.5 mg: Cases: 8 (14.5%) Control: 7 (12.1%) 45 mg: Cases: 8 (13.8%) Control: 7 (12.1%).	Cases: Seizures: 2 (3% in treated with 22.5 mg at initial dose	Yes
Frank S, et al., 2016 [26]	Low	Cases: 55.4 (10.3) Control: 52.1 (13.4)	90	13	VMAT-2 (deutetrabenazine 6 mg, mg initial dose 48 mg target dose	UHDRS-TMC and TMS; PGIC; CGIC; SF-36; Berg Balance test	Cases: somnolence 5 (11.1) Control: irritability 6 (13.3%)	Cases: cholecystitis: 1 (2.2%), and agitated depression: 1 (2.2%) Control: COPD: 1 (2.2%)	Yes
de Yebenes JG, et al., 2011 [23]	Low	Cases: 51 (10.7) Control: 49.1 (9.6)	292	26	Dopamine stabilizer (pridopidine) 45 mg and 90 mg	UHDRS -mMS; CGI; UHDRS, Stroop test	45 mg: Cases: dizziness 11 (7%) Control: falls, chorea and nauseas: 8 (5.5%) 90 mg: Cases: falls 13 (9%) Control: falls, chorea, and nausea: 8 (5.5%)	NA	Yes
Stimming EF, et al., 2023 [14]	Low	Cases: 54.1 (10.1) Control: 53.3 (11.4)	125	14 weeks	VMAT-2 (deutetrabenazine 40 mg, mg initial dose 80 mg target dose	UHDRS- TMC; CGIC; PGI; Neuro-Qol	Cases: somnolence: 5 (16%) Control: falls: 8 (13%)	Angioedema: 1 (2%) in cases group, colon cancer: 1 (2%), and psychosis: 1 (2%) in control group	Yes
Marshall F, et al., 2006 [21]	Low	Cases: 49.4 (12.4) Control: 48.8 (10.5)	84	12 weeks	VMAT-2 (deutetrabenazine) 12.5 mg, mg initial dose 100 mg target dose	UHDRS, HAM-D, CGI, UPDRS, Stroop test, TE,	Fatigue Cases: 7 (14.3%) Control: 2 (6.9%)	Multiples: 4 (7.4%) in cases group	Yes

All studies are randomized, multinational, placebo-controlled trials [14,21,22,23,24,25,26]. UHDRS: Unified Huntington’s Disease Rating Scale; HAM-D: Hamilton Depression Scale; CGI: Clinical Global Impression; UPDRS: Unified Parkinson’s Disease Rating Scale; TE: treatment effect; mMS: modified motor scale; PGI: Patient Global Impression; Neuro-Qol: Quality of Life in Neurological Disorders; CGIC: Clinical Global Impression Change; PGIC: Patient Global Impression Change; SF-36: 36-Item Short Form Health Survey; HADS: Hospital Anxiety and Depression Scale; TMS: total motor score; TFC: Total Functional Capacity, NA: Information not available.

## Data Availability

Data sharing is not applicable to this article as no datasets were generated or analyzed during the study.

## Supplementary Materials

The following supporting information can be downloaded at: https://www.mdpi.com/article/10.3390/medsci13030201/s1, Table S1. PubMed searches; Table S2. Web of Science searches; Table S3. Embase searches; Table S4. CINAHL searches; Table S5. Scopus searches; Table S6. Cochrane searches; Table S7. CNKI searches; Table S8: Subgroup analysis by treatment country; Table S9: Subgroup analysis by year; Table S10: Subgroup analysis by risk of bias; Table S11: Subgroup analysis by study design; Table S12: Subgroup analysis by dosing escalation; Table S13: Subgroup analysis by treatment period; Table S14: Sensitivity analysis with leave-one-out method.

## References

[1] Bachoud-Lévi A.C., Ferreira J., Massart R., Youssov K., Rosser A., Busse M., Craufurd D., Reilmann R., De Michele G., Rae D. (2019). international guidelines for the treatment of Huntington’s disease. Front. Neurol..

[2] Medina A., Mahjoub Y., Shaver L., Pringsheim T. (2022). Prevalence and Incidence of Huntington’s Disease: An Updated Systematic Review and Meta-Analysis. Mov. Disord..

[3] Baig S.S., Strong M., Quarrell O.W. (2016). The global prevalence of Huntington’s disease: A systematic review and discussion. Neurodegener. Dis. Manag..

[4] Lee J.-M., Ramos E.M., Lee J.H., Gillis T.P., Mysore J.S., Hayden M.R., Warby S.C., Morrison P.J., A Nance M., A Ross C. (2012). CAG repeat expansion in Huntington disease determines age at onset in a fully dominant fashion. Neurology.

[5] Gil J.M., Rego A.C. (2008). Mechanisms of neurodegeneration in Huntington’s disease. Eur. J. Neurosci..

[6] Kim A., Lalonde K., Truesdell A., Gomes Welter P., Brocardo P.S., Rosenstock T.R., Gil-Mohapel J. (2021). New Avenues for the Treatment of Huntington’s Disease. Int. J. Mol. Sci..

[7] Chen S., Liang T., Xue T., Xue S., Xue Q. (2021). Pridopidine for the Improvement of Motor Function in Patients With Huntington’s Disease: A Systematic Review and Meta-Analysis of Randomized Controlled Trials. Front. Neurol..

[8] Geva M., Kusko R., Soares H., Fowler K.D., Birnberg T., Barash S., Wagner A.M., Fine T., Lysaght A., Weiner B. (2016). Pridopidine activates neuroprotective pathways impaired in Huntington Disease. Hum. Mol. Genet..

[9] Grachev I.D., Meyer P.M., Becker G.A., Bronzel M., Marsteller D., Pastino G., Voges O., Rabinovich L., Knebel H., Zientek F. (2021). Sigma-1 and dopamine D2/D3 receptor occupancy of pridopidine in healthy volunteers and patients with Huntington disease: A [18F] fluspidine and [18F] fallypride PET study. Eur. J. Nucl. Med. Mol. Imaging.

[10] Koch J., Shi W.X., Dashtipour K. (2020). VMAT2 inhibitors for the treatment of hyperkinetic movement disorders. Pharmacol. Ther..

[11] Waters S., Ponten H., Edling M., Svanberg B., Klamer D., Waters N. (2014). The dopaminergic stabilizers pridopidine and ordopidine enhance cortico-striatal Arc gene expression. J. Neural Transm..

[12] Frank S., Testa C., Edmondson M.C., Goldstein J., Kayson E., Leavitt B.R., Oakes D., O’neill C., Vaughan C., Whaley J. (2022). The Safety of Deutetrabenazine for Chorea in Huntington Disease: An Open-Label Extension Study. CNS Drugs.

[13] Vadlamani N., Ibrahimli S., Khan F.A., A Castillo J., Amaravadi K.S.S., Nalisetty P., Khan S. (2024). Efficacy and Safety of Tetrabenazine in Reducing Chorea and Improving Motor Function in Individuals With Huntington’s Disease: A Systematic Review. Cureus.

[14] Stimming E.F., O Claassen D., Kayson E., Goldstein J., Mehanna R., Zhang H., Liang G.S., Haubenberger D., Adams J., Beck C. (2023). Safety and efficacy of valbenazine for the treatment of chorea associated with Huntington’s disease (KINECT-HD): A phase 3, randomised, double-blind, placebo-controlled trial. Lancet Neurol..

[15] Page M.J., Moher D., Bossuyt P.M., Boutron I., Hoffmann T.C., Mulrow C.D., Shamseer L., Tetzlaff J.M., Akl E.A., Brennan S.E. (2021). PRISMA 2020 explanation and elaboration: Updated guidance and exemplars for reporting systematic reviews. BMJ.

[16] Ouzzani M., Hammady H., Fedorowicz Z., Elmagarmid A. (2016). Rayyan-a web and mobile app for systematic reviews. Syst. Rev..

[17] Risk of Bias 2 (RoB 2) Tool|Cochrane Methods. https://methods.cochrane.org/risk-bias-2.

[18] Harrer M., Cuijpers P., Furukawa T.A., Ebert D.D. (2021). Doing Meta-Analysis with R.

[19] Jackson D., White I.R., Thompson S.G. (2010). Extending DerSimonian and Laird’s methodology to perform multivariate random effects meta-analyses. Stat. Med..

[20] Thorlund K., Engstrøm J., Wetterslev J., Brok J., Imberger G., Gluud S. (2017). User Manual for Trial Sequential Analysis (TSA).

[21] Marshall F.J. (2006). Tetrabenazine as antichorea therapy in Huntington disease: A randomized controlled trial. Neurology.

[22] Reilmann R., McGarry A., Grachev I.D., Savola J.-M., Borowsky B., Eyal E., Gross N., Langbehn D., Schubert R., Wickenberg A.T. (2019). Safety and efficacy of pridopidine in patients with Huntington’s disease (PRIDE-HD): A phase 2, randomised, placebo-controlled, multicentre, dose-ranging study. Lancet Neurol..

[23] De Yebenes J.G., Landwehrmeyer B., Squitieri F., Reilmann R., Rosser A., Barker R.A., Saft C., Magnet M.K., Sword A., Rembratt A. (2011). Pridopidine for the treatment of motor function in patients with Huntington’s disease (MermaiHD): A phase 3, randomised, double-blind, placebo-controlled trial. Lancet Neurol..

[24] Lundin A., Dietrichs E., Haghighi S., Göller M.-L., Heiberg A., Loutfi G., Widner H., Wiktorin K., Wiklund L., Svenningsson A. (2010). Efficacy and safety of the dopaminergic stabilizer pridopidine (ACR16) in patients with Huntington’s disease. Clin. Neuropharmacol..

[25] Kieburtz K., McGarry A., McDermott M.P., The Huntington Study Group HART Investigators (2013). A randomized, double-blind, placebo-controlled trial of pridopidine in Huntington’s disease. Mov. Disord..

[26] Frank S., Testa C.M., Stamler D., Kayson E., Davis C., Edmondson M.C., Kinel S., Leavitt B., Oakes D., Huntington Study Group (2016). Effect of Deutetrabenazine on Chorea Among Patients with Huntington Disease: A Randomized Clinical Trial. JAMA.

[27] Richardson K., McCusker E., Loy C., Griffith J., Mills J., Paulsen J. (2010). Poster 18: Lack of Awareness of Motor and Cognitive Phenoconversion in Huntington’s Disease. Neurotherapeutics.

[28] Asla M.M., Nawar A.A., Abdelsalam A., Elsayed E., Rizk M.A., Hussein M.A., Kamel W.A. (2022). The Efficacy and Safety of Pridopidine on Treatment of Patients with Huntington’s Disease: A Systematic Review and Meta-Analysis. Mov. Disord. Clin. Pract..

[29] Connolly A., Wallman P., Dzahini O., Howes O., Taylor D. (2024). Meta-analysis and systematic review of vesicular monoamine transporter (VMAT-2) inhibitors in schizophrenia and psychosis. Psychopharmacology.

[30] Stahl S.M. (2018). Mechanism of action of vesicular monoamine transporter 2 (VMAT2) inhibitors in tardive dyskinesia: Reducing dopamine leads to less ‘go’ and more ‘stop’ from the motor striatum for robust therapeutic effects. CNS Spectr..

[31] Goldberg Y.P., Navon-Perry L., Cruz-Herranz A., Chen K., Hecker-Barth G., Spiegel K., Cohen Y., Niethammer M., Tan A.M., Schuring H. (2025). The Safety Profile of Pridopidine, a Novel Sigma-1 Receptor Agonist for the Treatment of Huntington’s Disease. CNS Drugs.

