# Supplementary material for: Efficacy and Safety of VMAT-2 Inhibitors and Dopamine Stabilizers for Huntington’s Chorea: A Systematic Review, Meta-Analysis, and Trial Sequential Analysis
Source: Med Sci (Basel). 2025 Sep 22;13(3):201. doi: 10.3390/medsci13030201 (PMC12471964; doi:10.3390/medsci13030201)
Supplement: Supplementary file 1 [file medsci-13-00201-s001.zip › medsci-3825238-supplementary.pdf]

Supplementary material

Table S1. PubMed searches

PubMed	
Search	Results
(((Huntington Disease[Mesh]) OR (Huntington chorea[Title/Abstract]) OR (Huntington's chorea[Title/Abstract]) OR (Huntington disease[Title/Abstract]) OR (Huntington's disease[Title/Abstract]) OR (HD[Title/Abstract]))<br><br>AND<br><br>((Tetrabenazine[Mesh]) OR (Tetrabenazine[Title/Abstract]) OR (Valbenazine[Title/Abstract]) OR (Deutetrabenazine[Title/Abstract]) OR (Pridopidine[Title/Abstract]) OR (VMAT2 inhibitor[Title/Abstract]) OR (VMAT2 inhibitors[Title/Abstract]) OR (dopamine stabilizer[Title/Abstract]) OR (dopamine stabilizers[Title/Abstract]) OR (dopamine stabilizing drug[Title/Abstract]) OR (dopamine stabilizing drugs[Title/Abstract]) OR (dopaminergic stabilizer[Title/Abstract]) OR (dopaminergic stabilizers[Title/Abstract]) OR (Ordopidine[Title/Abstract]) OR (Pridopidine[Title/Abstract])))	329

Table S2. Web of Science searches

Web of Science	
Search	Results
((ALL= ("Huntington Disease") OR ALL= ("Huntington chorea") OR ALL= ("Huntington's chorea") OR ALL= ("Huntington disease") OR ALL= ("Huntington's disease") OR ALL=("HD")))	444

AND ((ALL=("Tetrabenazine") OR ALL=("Valbenazine") OR ALL=("Deutetrabenazine") OR ALL=("Pridopidine") OR ALL=("VMAT2 inhibitor") OR ALL=("VMAT2 inhibitors") OR ALL=("dopamine stabilizer") OR ALL=("dopamine stabilizers") OR ALL=("dopamine stabilizing drug") OR ALL=("dopamine stabilizing drugs") OR ALL=("dopaminergic stabilizer") OR ALL=("dopaminergic stabilizers") OR ALL=("Ordopidine") OR ALL=("Pridopidine")))	

Table S3. Embase searches

Embase	
Search	Results
('huntington disease'/exp OR 'huntington chorea':ti,ab OR 'huntington choreas':ti,ab OR 'huntington disease':ti,ab OR 'huntington diseases':ti,ab OR 'hd':ti,ab)<br><br>AND<br><br>('tetrabenazine'/exp OR 'valbenazine':ti,ab OR 'deutetrabenazine':ti,ab OR 'pridopidine':ti,ab OR 'vmat2 inhibitor':ti,ab OR 'vmat2 inhibitors':ti,ab OR 'dopamine stabilizer':ti,ab OR 'dopamine stabilizers':ti,ab OR 'dopamine stabilizing drug':ti,ab OR 'dopamine stabilizing drugs':ti,ab OR 'dopaminergic stabilizer':ti,ab OR 'dopaminergic stabilizers':ti,ab OR 'ordopidine':ti,ab)	1083

Table S4. CINAHL searches

CINAHL	
Search	Results

(((MH "Huntington Disease" OR TX "Huntington chorea" OR TX "Huntington's chorea" OR TX "Huntington disease" OR TX "Huntington's disease" OR TX "HD"))) AND (((MH "Tetrabenazine" OR TX "Tetrabenazine" OR TX "Valbenazine" OR TX "Deutetrabenazine" OR TX "Pridopidine" OR TX "VMAT2 inhibitor" OR TX "VMAT2 inhibitors" OR TX "dopamine stabilizer" OR TX "dopamine stabilizers" OR TX "dopamine s CINAHL: stabilizing drug" OR TX "dopamine stabilizing drugs" OR TX "dopaminergic stabilizer" OR TX "dopaminergic stabilizers" OR TX "Ordopidine" OR TX "Pridopidine")))	115

Table S5. Scopus searches

Scopus	
Search	Results
(TITLE-ABS-KEY("Huntington disease") OR TITLE-ABS-KEY("Huntington chorea") OR TITLE-ABS-KEY ("Huntington's chorea") OR TITLE-ABS-KEY("Huntington's disease") OR TITLE-ABS-KEY("Huntington diseases") OR TITLE-ABS-KEY("HD"))<br><br>AND (TITLE-ABS-KEY("Tetrabenazine") OR TITLE-ABS-KEY("Valbenazine") OR TITLE-ABS-KEY("Deutetrabenazine") OR TITLE-ABS-KEY("Pridopidine") OR TITLE-ABS-KEY("VMAT2 inhibitor") OR TITLE-ABS-KEY("VMAT2 inhibitors") OR TITLE-ABS-KEY("dopamine stabilizer") OR TITLE-ABS-KEY("dopamine stabilizers") OR TITLE-ABS-KEY("dopamine stabilizing drug") OR TITLE-ABS-KEY("dopamine stabilizing drugs") OR TITLE-ABS-KEY("dopaminergic stabilizer") OR TITLE-ABS-KEY("dopaminergic stabilizers") OR TITLE-ABS-KEY("Ordopidine"))	886

Table S6. Cochrane searches

Cochrane		
Search		Results
1	MeSH descriptor: [Huntington Disease] explode all trees	351
2	("Huntington chorea":ti,ab OR "Huntington's chorea":ti,ab OR "Huntington disease":ti,ab OR "Huntington's disease":ti,ab OR "Huntington diseases":ti,ab OR "HD":ti,ab)	8772
3	MeSH descriptor: [Tetrabenazine] explode all trees	82
4	("Tetrabenazine":ti,ab OR "Valbenazine":ti,ab OR "Deutetrabenazine":ti,ab OR "Pridopidine":ti,ab OR "VMAT2 inhibitor":ti,ab OR "VMAT2 inhibitors":ti,ab OR "dopamine stabilizer":ti,ab OR "dopamine stabilizers":ti,ab OR "dopamine stabilizing drug":ti,ab OR "dopamine stabilizing drugs":ti,ab OR "dopaminergic stabilizer":ti,ab OR "dopaminergic stabilizers":ti,ab OR "Ordopidine":ti,ab)	313
5	#1 OR #	8797
6	#3 OR #4	329
7	#5 AND #6	140

Table S7. CNKI searches

CNKI	Results
((SU=("Huntington disease" + "Huntington chorea" + "Huntington's chorea" + "Huntington's disease" + "Huntington diseases" + "HD"))	1290

AND<br><br>(TKA=("Tetrabenazine" + "Valbenazine" + "Deutetrabenazine" + "Pridopidine" + "VMAT2 inhibitor" + "VMAT2 inhibitors" + "dopamine stabilizer" + "dopamine stabilizers" + "dopamine stabilizing drug" + "dopamine stabilizing drugs" + "dopaminergic stabilizer" + "dopaminergic stabilizers" + "Ordopidine")))	

Table S8: Subgroup analysis by treatment country

**Variable**	**Subgroup**	**k**	**RR (95% CI)**	**I² (%)**	**P-value (Between Groups)**
Country	Multicenter	**4**	-0.9815 (-2.4767, 0.5137)	--	--

Table S9: Subgroup analysis by year

**Variable**	**Subgroup**	**k**	**RR (95% CI)**	**I² (%)**	**P-value (Between Groups)**
Year	2018	1	1.1025 (-1.2371, 3.4421)	--	
	2010	1	-1.7000 (-4.9983, 1.5983)	--	

	2013	1	-1.3846 (-4.0388, 1.2696)	--	
	2011	1	-1.9043 (-3.6250, -0.1836)	--	
					**0.2206**

Table S10: Subgroup analysis by risk of bias

**Variable**	**Subgroup**	**k**	**RR (95% CI)**	**I² (%)**	**P-value (Between Groups)**
Risk of Bias	Low	4	-0.9815 (-2.4767, 0.5137)	--	--

Table S11: Subgroup analysis by study design

**Variable**	**Subgroup**	**k**	**RR (95% CI)**	**I² (%)**	**P-value (Between Groups)**
Study design	RCT	4	-0.9815 (-2.4767, 0.5137)	--	--

Table S12: Subgroup analysis by dosing escalation

Variable	Subgroup	k	RR (95% CI)	I² (%)	p-value (Between Groups)
Dosing escalation	NO	3	-0.8392 (-2.8485, 1.1700)	--	
	YES	1	-1.3846 (-4.0388, 1.2696)	--	
					**0.7481**

Table S13: Subgroup analysis by treatment period

Variable	Subgroup	k	RR (95% CI)	I² (%)	P-value (Between Groups)
Treatment period	>26 weeks	1	1.1025 (-1.2371, 3.4421)	--	
	≤26 weeks	3	-1.7424 (-3.0650, -0.4198)	0.0%	
					**0.0380**

Table S14: Sensitivity analysis with leave-one-out method

Author	Effect (95% CI:	I²	DFFITS	Cook's D	QE (del)	Is Influential

	**Lower; Upper)**					
Omitting Reilmann R, et al. 2018	-1.742 (95% CI: -3.065; -0.420)	0.000	1.1729	1.3756	0.1045	Yes
Omitting Lundin A, et al. 2010	-0.964 (95% CI: -2.192; 0.265)	0.528	-0.1527	0.0233	4.2406	No
Omitting Kieburtz K, et al. 2013	-0.977 (95% CI: -2.255; 0.301)	0.539	-0.1307	0.0171	4.3350	No
Omitting de Yebenes JG, et al. 2011	-0.363 (95% CI: -1.913; 1.186)	0.261	-1.1745	1.3793	2.7076	Yes
